# Numerical Considerations in the Modeling of a High Explosive Cylinder Experiment Using an ALE Continuum Mechanics Code

**DOI:** 10.3390/ma13194448

**Published:** 2020-10-07

**Authors:** Marvin A. Zocher, Tariq D. Aslam

**Affiliations:** Los Alamos National Laboratory, Los Alamos, NM, 87545, USA; aslam@lanl.gov

**Keywords:** high explosive cylinder, reactive burn model, finite volume method

## Abstract

A series of experiments involving the detonation of PBX 9501 encased in a copper cylinder are modeled with the objective of evaluating a proposed set of phenomenological parameters for the Wescott–Stewart–Davis reactive burn model. The numerical analysis is conducted using the Los Alamos continuum mechanics code FLAG. Numerical considerations pertaining to various aspects of modeling the experiments using FLAG are discussed. It is shown that use of the proposed set of phenomenological parameters results in predictions of free-surface velocity that match empirically measured velocities reasonably well.

## 1. Introduction

The high explosive (HE) cylinder test is a tool that is often used for the purpose of investigating reactive burn and the constitutive response of HE detonation products and for validating numerical models thereof. The test normally invloves the initiation of detonation at one end of a cylinder of HE that is encased circumferentially in a thin metallic cylindrical sleeve (the metallic sleeve is open-ended). As the detonation wave propagates through the length of the cylinder, the metallic confinement expands. By carefully measuring this expansion, one may gain valuable insight into the constitutive behavior of the detonation products, and can use the measurements of cylinder expansion for quantitative model validation, comparing predicted expansion to that which is measured.

**Figure 1:**
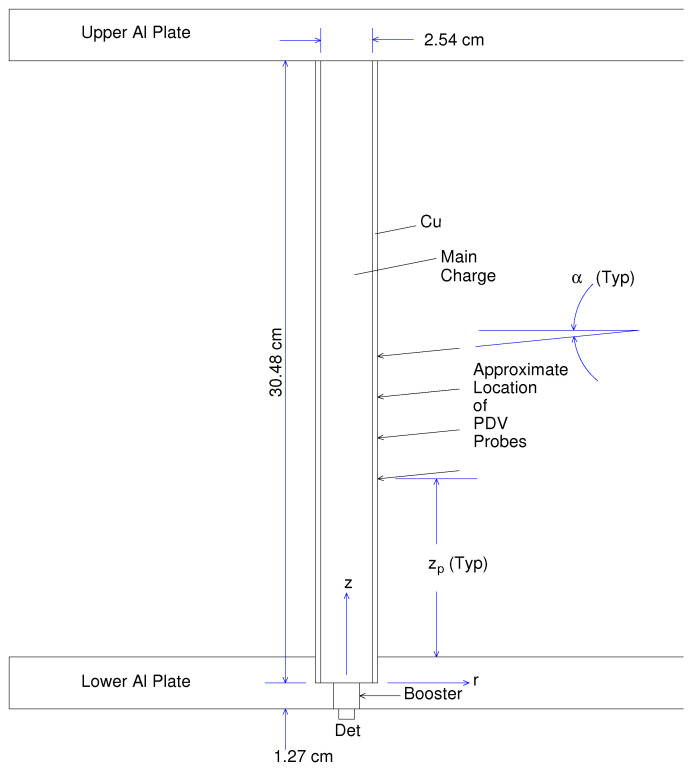
Experimental assembly.

*Circa* 2011, Pemberton et al. [1] conducted a series of cylinder expansion experiments involving PBX 9501 encased in copper. A diagram of the experimental assembly is provided in Figure 1. The diameter of the encased HE was 2.54 cm and the wall thickness of the copper cylinder was 0.254 cm. The length of the composite HE/Cu cylinder was 30.48 cm. Detonation in the PBX 9501 main charge was initiated by a PBX 9407 booster and an RP-1 detonator. The diameter of the booster was 1.27 cm and that of the explosive component of the RP-1 was 0.762 cm. The detonator end of the composite cylinder sat within a 1.27 cm deep indentation cut into the lower aluminum plate. The opposite end sat flush against a second aluminum plate. Six shots were conducted, however three of those shots (shots 2, 3, and 5) suffered from assembly issues that resulted in significant gaps between explosive and copper. We therefore restricted our study to the modeling of shots 1, 4, and 6.

Cylinder expansion was recorded using Photon Doppler Velocimetry (PDV). Measurements were taken at eight different locations with variation in both axial and azimuthal coordinate. Note that we shall ignore herein variation in the azimuthal coordinate since, as will be discussed later, our numerical modeling of the experiment was conducted assuming 2d-axisymmetry and therefore azimuth has no meaning in our analysis. Note also that, as suggested in Figure 1, the line of sight of the PDV probes is not normal to the axis of the composite cylinder, but is canted at a small angle, α, relative to the normal. In Table 1 we provide the axial coordinate and value of α for each of the 8 PDV spot locations (spot “illuminated” on the surface of the Cu cylinder).

In the current work, three of the experiments described above (shots 1, 4, and 6) are modeled using the Los Alamos finite volume continuum mechanics arbitrary Lagrangian–Eulerian (ALE) code: FLAG [2]. The ultimate objective of that effort is the evaluation of a set of phenomenological parameters for the Wescott–Stewart–Davis (WSD) reactive burn model [3]. Our focus herein is to report on the numerical modeling of the experiment, not on the WSD model, per se. Consequently, we will not go into much detail concerning WSD, but shall, instead, refer the reader to [3] and will focus our attention herein on our modeling exercise only.

The use of numerical methods (in particular continuum mechanics codes) for modeling the HE cylinder test is not new, having been practiced now for well over 50 years. Over that time a wide variety of numerical methodologies have been developed and employed, among which may be included: (a) finite difference, finite element, finite volume, and smooth particle hydrodynamics (SPH) methods, (b) Eulerian, Lagrangian, ALE, and meshless approaches, and (c) both explicit and implicit time integration schemes. We shall not provide the reader with a review of the extensive literature, but shall, instead, point to a very small number of examples wherein a continuum mechanics code was used to model the cylinder test, often for the purpose of phenomenological model validation. In perhaps one of the earliest efforts, Wilkins [4] used a two-dimensional Lagrangian finite difference code (HEMP) to determine the equation of state (EOS) at low pressures for the HE detonation products. Tang and Scannapieco [5] employed an Eulerian code with an adaptive mesh refinement (AMR) capability in a study of the dynamics of cylinder expansion while noting that, in general, a primary objective of the HE cylinder test is the extraction of EOS information. Elek et al. [6] employed a commercial finite element code (Abaqus) and coupled Eulerian Lagrangian (CEL) methodologies in a determination of detonation product EOS. Yang et al. [7] developed a meshless and Lagrangian SPH approach termed modified cylindrical smoothed particle hydrodynamics (MCSPH) and compared results obtained using MCSPH to those obtained using the LS-DYNA code and ALE. It is not our objective to compare the capabilities of the FLAG code to any other, nor is it to argue for the primacy of one numerical methodology over another. Rather, our aim is much more limited, merely a preliminary validation of a set of WSD parameters based upon use of the numerical models in FLAG. To our knowledge, this publication will be among some of the earliest publications wherein the HE cylinder test has been modeled using the WSD model. However, we must note that we are not the first to conduct such analysis, for we are aware of some earlier unpublished work by Von Whitley [8] wherein he used WSD in this manner. We might also mention some recently published work by Price et al. [9] wherein cylinder expansion tests were modeled for the purpose of evaluating an Arrhenius WSD (AWSD) model for PBX 9502, and by Zocher et al. [10] wherein both cylinder expansion tests and slab expansion tests were modeled using a WSD model for PBX 9501. In the following sections we very briefly describe the WSD model as programmed into FLAG, briefly discuss our modeling of the experiment, and finally, conclude with an assessment as to the efficacy of our modeling approach.

## 2. HE Constitutive and Reactive Burn Models Plus the Modeling of Cu

Within FLAG it is customary to use the WSD reactive burn model in concert with a two-part EOS for the HE. In this case we have chosen to use Davis-type EOSs [3] for both reactants and products.

### 2.1. Davis-Type EOS for Reactants

Wescott et. al [3] gives equations for p=p(v,E) and T=T(v,E) where *p*, *T*, *v*, and *E* denote pressure, temperature, specific volume, and specific internal energy, respectively. We can equivalently express equations for *p* and *T* in terms of density, ρ, and specific internal energy (here we will use *e* instead of *E* to denote specific internal energy), that is, p=p(ρ,e) and T=T(ρ,e). Doing so yields:

(1) $$p\left(\rho ,e\right)=p_{s}\left(\rho \right)+\rho \;\Gamma \left(\rho \right)\left[e-e_{s}\left(\rho \right)\right]$$

(2) $$T\left(\rho ,E\right)=T_{s}\left(\rho \right){\left\{\frac{1+\alpha_{st}}{C_{v0}T_{s}\left(\rho \right)}\left[(e-e_{s}\left(\rho \right)\right]+1\right\}}^{\frac{1}{1+\alpha_{st}}}$$

where,

(3) $$p_{s}\left(\rho \right)=\left\{\begin{array}{cc} \hat{p}\left[\sum_{j=1}^{3}\frac{{\left(4By\right)}^{j}}{j!}+\frac{C{\left(4By\right)}^{4}}{4!}+\frac{y^{2}}{{\left(1-y\right)}^{4}}\right]\; & \mathrm{for}\;\rho \geq \rho_{0}\\ \hat{p}\left[\exp\left(4By\right)-1\right]\; & \mathrm{for}\;\rho <\rho_{0} \end{array}\right.$$

(4) $$e_{s}\left(\rho \right)=\frac{1}{\rho_{0}}\int_{0}^{y}p_{s}\left(\xi \right)\;d\xi$$

(5) $$\Gamma \left(\rho \right)=\left\{\begin{array}{cc} \Gamma_{0}+Zy & \mathrm{for}\;\rho \geq \rho_{0}\;\\ \Gamma_{0} & \mathrm{for}\;\rho <\rho_{0} \end{array}\right.$$

(6) $$\hat{p}=\frac{\rho_{0}A^{2}}{4B}$$

(7) $$y=1-\frac{\rho_{0}}{\rho }$$

(8) $$T_{s}\left(\rho \right)=\left\{\begin{array}{cc} T_{0}\;e^{-Zy}{\left(\frac{\rho_{0}}{\rho }\right)}^{-(\Gamma_{0}+Z)}\; & \mathrm{for}\;\rho \geq \rho_{0}\;\\ T_{0}{\left(\frac{\rho_{0}}{\rho }\right)}^{-\Gamma_{0}}\; & \mathrm{for}\;\rho <\rho_{0}\; \end{array}\right.$$

In Equations (1)–(8), $e_{s}$, $p_{s}$, and $T_{s}$ are the specific internal energy, pressure, and temperature on the isentrope passing through reference density, $\rho_{0}$, respectively, Γ is the Grüneisen gamma, and *A*, *B*, *C*, $\alpha_{st}$, $C_{v0}$, $\Gamma_{0}$, $v_{0}$, $T_{0}$, *Z* are model parameters. Note that the term $E_{0}$ appearing in Equation (26) of reference [3] is omitted from our Equation (4) (the term corresponding to $E_{0}$ ($e_{0}$ in our notation) is handled elsewhere in FLAG; see Equation (12)). The Davis reactants parameter set used in this work is given in Table 2.

### 2.2. Davis-Type EOS for Products

As was the case for the reactants EOSs, the Davis-type EOSs for products as delineated in [3] is given in in terms of specific volume and specific energy, that is, p=p(v,E) and T=T(v,E). Moreover, as was done for reactants, we shall equivalently express equations for *p* and *T* in terms of density and specific internal energy (and as in the previous section we shall use *e* to denote specific internal energy as opposed to *E*, which was used in Reference [3]).

(9) $$p\left(\rho ,e\right)=p_{s}\left(\rho \right)+\rho \;\Gamma \left(\rho \right)\left[e-e_{s}\left(\rho \right)\right]$$

(10) $$T\left(\rho ,e\right)=T_{s}\left(\rho \right)+\frac{e-e_{s}\left(\rho \right)}{C_{v}}$$

where,

(11) $$p_{s}\left(\rho \right)=p_{c}\;\frac{{\left[\frac{1}{2}{\left(\frac{1}{\rho v_{c}}\right)}^{n}+\frac{1}{2}{\left(\frac{1}{\rho v_{c}}\right)}^{-n}\right]}^{a/n}}{{\left(\frac{1}{\rho v_{c}}\right)}^{k+a}}\;\frac{\left(k-1+F(\rho )\right)}{\left(k-1+a\right)}$$

(12) $$e_{s}\left(\rho \right)=e_{c}\;\frac{{\left[\frac{1}{2}{\left(\frac{1}{\rho v_{c}}\right)}^{n}+\frac{1}{2}{\left(\frac{1}{\rho v_{c}}\right)}^{-n}\right]}^{a/n}}{{\left(\frac{1}{\rho v_{c}}\right)}^{k-1+a}}-e_{0}$$

(13) Γ(ρ)=k−1+(1−b)F(ρ)

(14) $$F\left(\rho \right)=\frac{2a{\left(\frac{1}{\rho v_{c}}\right)}^{-n}}{{\left(\frac{1}{\rho v_{c}}\right)}^{n}+{\left(\frac{1}{\rho v_{c}}\right)}^{-n}}$$

(15) $$e_{c}=\frac{p_{c}v_{c}}{k-1+a}$$

(16) $$T_{s}\left(\rho \right)=T_{c}\;\frac{{\left[\frac{1}{2}{\left(\frac{1}{\rho v_{c}}\right)}^{n}+\frac{1}{2}{\left(\frac{1}{\rho v_{c}}\right)}^{-n}\right]}^{\left(a/n)(1-b\right)}}{{\left(\frac{1}{\rho v_{c}}\right)}^{k-1+a(1-b)}}$$

(17) $$T_{c}=\left[\frac{2^{-ab/n}}{k-1+a}\right]\frac{p_{c}v_{c}}{C_{v}}$$

In Equations (9)–(17) *a*, *b*, *k*, *n*, $p_{c}$, $v_{c}$, $C_{v}$, and $e_{0}$ are model parameters. The Davis products parameter set used in this work is given in Table 3.

### 2.3. WSD Reactive Burn Model

The reactive burn model as delineated in reference [3] consists of the following:

(18) $$r=r_{{}_{I}}S_{I}\left(\lambda \right)+r_{{}_{IG}}W\left(\rho_{{}_{SH}}\right)S_{G}\left(\lambda \right)+r_{{}_{DG}}\left[1-W(\rho_{{}_{SH}})\right]S_{G}\left(\lambda \right)+r_{{}_{B}}\left[1-S_{G}\left(\lambda \right)\right]$$

where,

(19) $$r_{{}_{I}}=k_{{}_{I}}\;{\left(\frac{\rho }{\rho_{0}}-1-a\right)}^{7}\;{\left(1-\lambda \right)}^{2/3}\;H\left(\frac{\rho }{\rho_{0}}-1-a\right)$$

(20) $$r_{{}_{IG}}=k_{{}_{IG}}{\left(\frac{p}{p_{{}_{CJ}}}\right)}^{4.5}\;\lambda^{1/3}\;\left(1-\lambda \right)$$

(21) $$r_{{}_{DG}}=k_{{}_{DG}}{\left(\frac{p}{p_{{}_{CJ}}}\right)}^{2}\;\lambda^{1/3}\;\left(1-\lambda \right)$$

(22) $$r_{{}_{B}}=k_{{}_{B}}\;\left(\frac{p}{p_{{}_{CJ}}}\right)\;{\left(1-\lambda \right)}^{1/2}$$

(23) $$S_{I}\left(\lambda \right)=\frac{1}{2}\left\{1-\tanh\left[200(\lambda -0.025)\right]\right\}$$

(24) $$S_{G}\left(\lambda \right)=\frac{1}{2}\left\{1-\tanh\left[30(\lambda -0.9)\right]\right\}$$

(25) $$W\left(\rho_{{}_{SH}}\right)=\frac{1}{2}\left\{1-\tanh\left[50\left(\frac{\rho_{{}_{SH}}}{\rho_{c}}-1\right)\right]\right\}$$

and where λ is the mass fraction of the detonation products, $\rho_{SH}$ is the shock density, and $k_{{}_{I}}$, $k_{{}_{IG}}$, $k_{{}_{DG}}$, $k_{{}_{B}}$, *a* and $\rho_{c}$ are model parameters. *H* denotes, of course, the Heaviside step function. We shall find it convenient to express the WSD Equation set (18)–(25) in a precisely equivalent form as:

(26) $$R=R_{{}_{I}}+R_{{}_{IG}}+R_{{}_{DG}}+R_{{}_{B}}$$

where,

(27) $$\begin{array}{c} R_{{}_{I}}\equiv r_{{}_{I}}S_{I}\left(\lambda \right)\\ R_{{}_{IG}}\equiv r_{{}_{IG}}W\left(\rho_{{}_{SH}}\right)S_{G}\left(\lambda \right)\\ R_{{}_{DG}}\equiv r_{{}_{DG}}\left[1-W(\rho_{{}_{SH}})\right]S_{G}\left(\lambda \right)\\ R_{{}_{B}}\equiv r_{{}_{B}}\left[1-S_{G}\left(\lambda \right)\right] \end{array}$$

It is important to note that Equations (19)–(25) were developed in reference [3] for a particular explosive (PBX 9502). We would like to generalize the WSD equation set so that it is potentially applicable to many explosives. We shall accomplish this by exchanging various numeric values that appear in Equations (19)–(25) with variables. Though not required, we shall also, since this is done in FLAG, replace ${\left(p/p_{{}_{CJ}}\right)}^{\mathrm{constant}}$ with ${\left(p\right)}^{\mathrm{variable}}$ and absorb ${\left(1/p_{{}_{CJ}}\right)}^{\mathrm{variable}}$ into user-settable model parameters. Doing so, the terms appearing in Equation (27) are expressed in a more general form as follows:

(28) $$R_{{}_{I}}=r_{{}_{i}}\;{\left(\frac{\rho }{\rho_{0}}-1-r_{{}_{a}}\right)}^{r_{{}_{x}}}\;{\left(1-\lambda \right)}^{r_{{}_{b}}}\;H\left(\frac{\rho }{\rho_{0}}-1-r_{{}_{a}}\right)\frac{1}{2}\left[1-\tanh\left\{200(\lambda -0.025)\right\}\right]$$

(29) $$R_{{}_{IG}}=r_{g1}{\left(1-\lambda \right)}^{r_{c}}\lambda^{r_{d}}p^{r_{y}}\frac{1}{2}\left[1-\tanh\left\{30\left(\lambda -r_{\mathrm{switch}}\right)\right\}\right]\frac{1}{2}\left[1-\tanh\left\{50\left(\frac{\rho_{{}_{SH}}}{\rho_{c}}-1\right)\right\}\right]$$

(30) $$R_{{}_{DG}}=r_{k}{\left(1-\lambda \right)}^{r_{c}}\lambda^{r_{d}}p^{r_{n}}\frac{1}{2}\left[1-\tanh\left\{30\left(\lambda -r_{\mathrm{switch}}\right)\right\}\right]\frac{1}{2}\left[1+\tanh\left\{50\left(\frac{\rho_{{}_{SH}}}{\rho_{c}}-1\right)\right\}\right]$$

(31) $$R_{{}_{B}}=r_{{}_{g2}}{\left(1-\lambda \right)}^{r_{{}_{e}}}p^{r_{{}_{z}}}\frac{1}{2}\left[1+\tanh\left\{30\left(\lambda -r_{\mathrm{switch}}\right)\right\}\right]$$

Equations (26) and (28)–(31) are what is programmed into FLAG. In these equations, $r_{a}$, $r_{b}$, $r_{c}$, $r_{d}$, $r_{e}$, $r_{i}$, $r_{k}$, $r_{n}$, $r_{x}$, $r_{y}$, $r_{z}$, $r_{g1}$, $r_{g2}$, $\rho_{0}$, $\rho_{c}$, and $r_{\mathrm{switch}}$ are model parameters. Additional model parameters that do not appear in Equations (28)–(31) include: rkdead (rate constant in preshock desensitization), rpign (minimum pressure to start burn), rpmax (maximum pressure at which to deaden), and rphel (Hugoniot elastic limit minimum to desensitize). These last four parameters are included for completeness, though they are not used in our analysis since rkdead is set to zero. The interested reader is directed to Wescott, Stewart, and Davis [11] for more on rkdead, rpign, rpmax, and rphel. The WSD parameter set used in this work is given in Table 4.

It is worth noting that for the parameter set shown in Table 4, the WSD burn model simplifies drastically from that shown in Equations (26) and (28)–(31). In fact, since $r_{i}$ and $r_{d}$ are 0, and $r_{b}$ = $r_{c}$ = $r_{e}$, and $r_{n}$ = $r_{y}$ = $r_{z}$, and $r_{g1}$ = $r_{g2}$ = $r_{k}$, the reaction rate simplifies (as given in [12]) to:

(32) $$R=a{\left(1-\lambda \right)}^{\nu }{\left(\frac{p}{B}\right)}^{N_{p}}$$

where *a*, ν, *B*, and $N_{p}$ are model parameters.

The reaction rate Equation (32) can be generalized to include an Arrhenius term (commonly included when dealing with gaseous detonation). This was done, e.g., in [13] where the rate equation is expressed as:

(33) $$R=a{\left(1-\lambda \right)}^{\nu }{\left(\frac{p}{B}\right)}^{N_{p}}\exp\left(\frac{-\rho E}{p}\right)$$

where *E* is the activation energy.

For our purposes, it is the equation set (26) and (28)–(31) (and the parameter set shown in Table 4) that are pertinent since this more general form is that which is programmed into FLAG.

### 2.4. Material Models Used for Cu

The spherical part of the Cauchy tensor is modeled using a tabular SESAME EOS (SESAME 3336). Initial density is set to 8.937507 g/cm${}^{3}$. The deviatoric part of the Cauchy tensor is modeled using the model of Preston et al. [14].

## 3. Numerical Modeling

All numerical modeling was conducted using the Los Alamos finite volume continuum mechanics code FLAG. The experiments were modeled assuming 2d-axisymmetry. FLAG, which was originally structured within a Lagrangian framework, has had its capabilities greatly enhanced through the incorporation of ALE methodologies such as nodal relaxation. A number of nodal relaxation schemes were tried in the current work, including the cn relaxer [15] and the feasibility set relaxer [16]. However, even with the use of nodal relaxation, it should be stated up front that modeling this experiment in high fidelity and without the use of some rather gross simplifications has proven to be deceivingly complicated. Much of this section will be devoted to a discussion of some of those complications, of various approaches that might be taken in order to overcome those complications, and finally of simplifications that the modeler might, however grudgingly, accept out of a sense of practicality.

In preparation for that discussion, let us first consider what happens once the main charge ignites, and what implications there may be for the modeler. At first glance, one might suspect that embedding the “detonator end” of the composite HE/Cu cylinder into an aluminum plate will result in significant challenges for the modeler. That suspicion, as we shall see, is not without merit. Even cursory thought applied in this direction reveals the difficulty. As detonation proceeds along the length of the HE cylinder, the thin copper cylinder will undergo significant expansion that “sweeps” from the detonator-end of the cylinder to the opposite end. That expansion will cause the end that is embedded in the lower aluminum plate to slide within that plate (in a generally upward and outward direction), ultimately being pulled entirely from the plate, spilling detonation products into the surroundings. Prior to exiting the confines of the aluminum plate, as the copper cylinder slides, detonation products flow to fill what would otherwise be the creation of a void. Sliding of the copper cylinder resulting in the flow of detonation products, first into what would otherwise be a void, and later into the surrounding environment, poses significant challenges for the modeler using ALE methodologies. Another challenge arises from the formation of an aluminum jet at the Cu/Al interface as the HE/Cu cylinder pushes aluminum aside to make way for its expansion. In the following section we shall consider these difficulties in more detail. After that we will discuss other issues and numerical considerations that the modeler must consider in the simulation of this experiment.

### 3.1. Numerical Considerations Related to the Lower Aluminum Plate

#### 3.1.1. To Slide or Not to Slide

**Figure 2:**
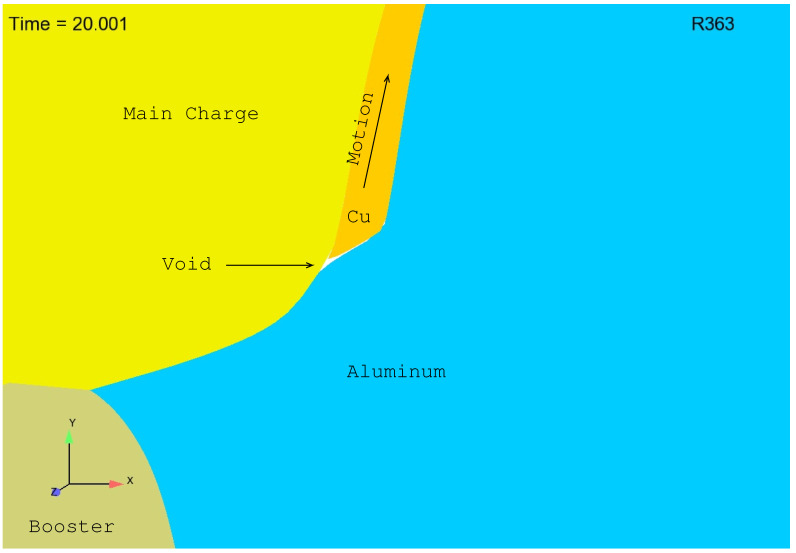
Initiation of a “void” at about 20 μs.

**Figure 3:**
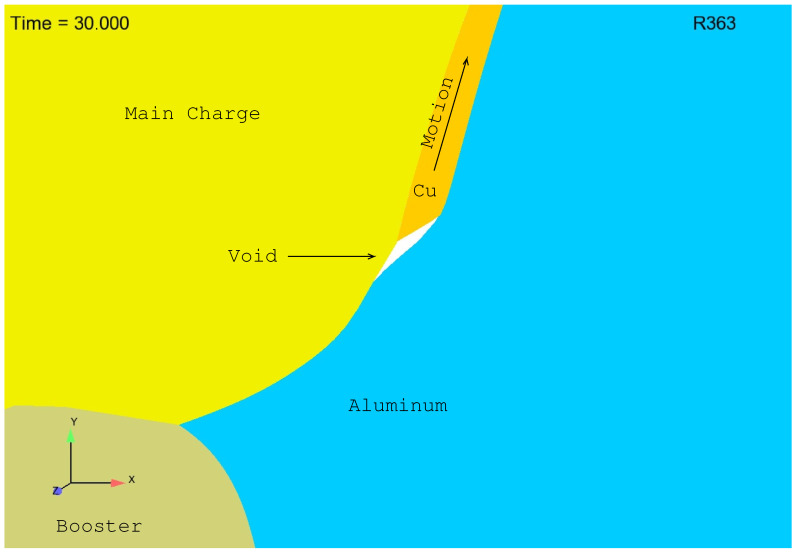
Larger “void” at 30 μs.

As described above, expansion of the copper cylinder naturally results in a sliding of the copper cylinder within the lower aluminum plate. Early stage sliding is illustrated in Figure 2 and Figure 3. Note that in these figures reference is made to R363, which is simply a “bookeeping” idintifier used by the authors to keep track of the many simulations conducted in the course of this study (over a thousand simulations were conducted and the results shown here are from simulation (or Run) number 363, i.e., R363). Note also that blue is aluminum, orange is copper, yellow is the main charge and the taupe color is the booster. In Figure 2 we see that the copper sleeve begins to slide within the aluminum plate at about 20 μs resulting in the formation of a “void”. In Figure 3 we see that at 30 μs the “void” has grown substantially. With the formation and growth of the “void”, detonation products naturally spill into this space filling what would otherwise be a true void. In the experiment this happens smoothly and instantaneously. In the simulation this spilling also occurs, but with it comes, as one might imagine, a propensity for tangling. The authors should note that the reason that the spilling of detonation products into the “void” is not seen in Figure 2 and Figure 3 is that the computational cells used in R363 are unrealistically large (too large to allow for the spilling of detonation products and far too large to result in realistic simulation of the actual experiment). The mesh of R363 was created, and R363 was conducted, merely for demonstration purposes: to illustrate “void” formation and growth. With a finer mesh, such as is absolutely required for accurately predicting thermophysical reality, one would see the computational cells rush to fill the “void” and, in the process, tangle.

As time passes, one should realize that the “void” discussed above will grow monotonically in size, eventually resulting in the spilling of detonation products into the surrounding environment when the copper cylinder escapes its aluminum confinement entirely. This may occur in the experiment with no deleterious consequences, however, the spilling of detonation products unencumbered into the environment poses a significant challenge for the numerical modeler.

One way to avoid the aforementioned difficulties associated with the spilling of detonation products into a “void” (and ultimately into the surrounding environment) would be to disallow separation of the copper cylinder from the aluminum plate at the “base” of the copper cylinder (along the nodes initially located at *z* = 0; see Figure 1). This would prevent the copper cylinder from sliding within the aluminum plate and opening up a “void”. However, this approach produces a new and different challenge. If the nodes at the “base” of the copper cylinder are not allowed to separate from the aluminum plate (separation occurs in the experiment), the copper cylinder will be artificially extruded in this region to a very small thickness. This extrusion, combined with curvilinearity, poses problems for the modeler in terms of aspect ratio and tangling within the copper cylinder. After trying numerous alternatives for dealing with problems associated with a sliding copper cylinder, it is this approach that the authors have most relied upon in modeling of this experiment.

#### 3.1.2. Dealing with an Aluminum Jet

**Figure 4:**
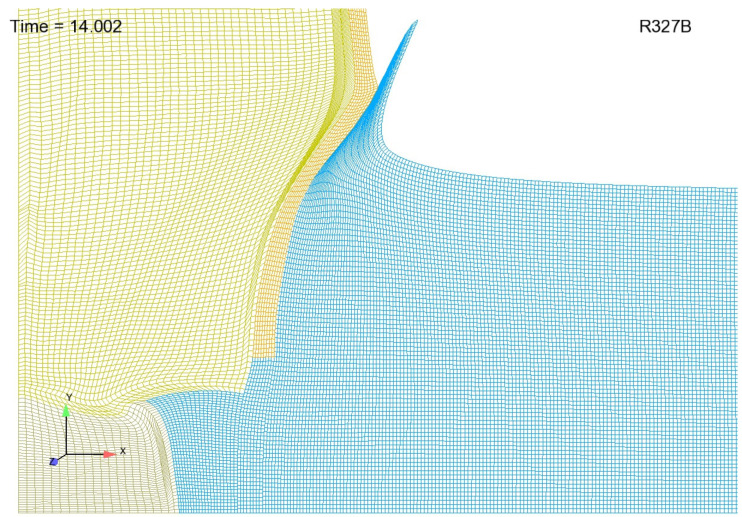
Jet morphology resultant from initially uniform mesh.

**Figure 5:**
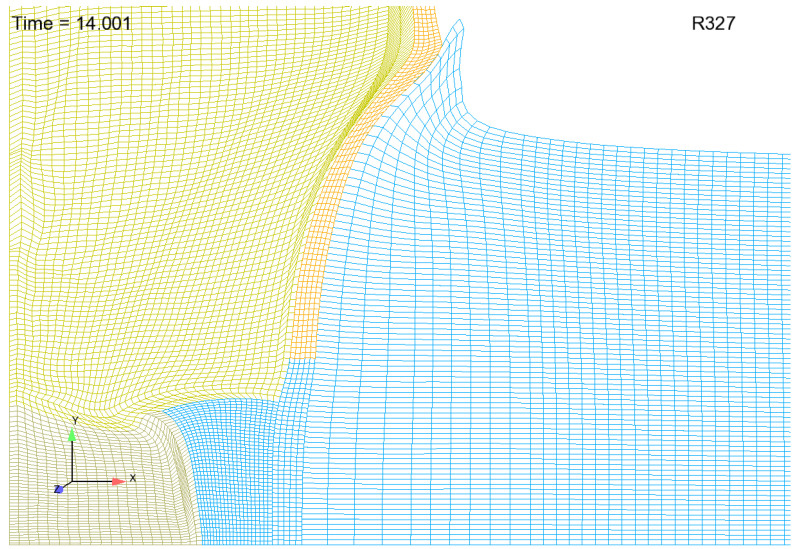
Jet morphology resultant from mesh employing a gradation tactic.

**Figure 6:**
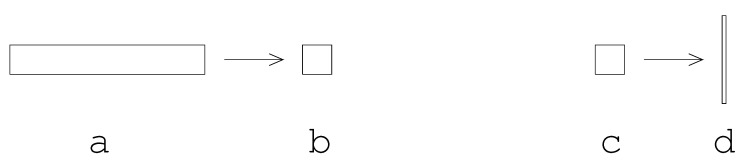
Meshing strategies in the aluminum near the copper: (**a**) and (**b**): graded strategy; (**c**) and (**d**): uniform strategy.

As mentioned earlier, an aluminum jet forms at the Cu/Al interface as the HE/Cu cylinder pushes aluminum aside to make way for its expansion. The image shown in Figure 4 depicts predicted jet morphology for a coarse mesh with initially uniform discretization within the aluminum. Note that the mesh density shown in Figure 4 is far too coarse for realistic simulation predictions. Moreover, with finer resolution, which is absolutely required for accurate predictions, the length of the jet greatly increases and its width greatly decreases, resulting in computational cells with very high aspect ratio that are prone to tangling. One possible approach to overcoming this difficulty is to employ nodal relaxation. However, with very fine meshes, nodal relaxation alone has proven to be insufficient in overcoming the aspect ratio and tangling problem. A supplementary tactic that can be employed in addition to nodal relaxation involves the creation of a mesh with a gradation in resolution involving computational cells in the aluminum near the copper that start off much “wider” than they are “tall”. Computational cells that are some distance away from the Copper can be meshed rather uniformly without incurring any difficulties. By taking this graded mesh approach, jet morphology, as depicted in Figure 4 is replaced by that depicted in Figure 5. This approach trades inaccuracies in jet morphology for a more robust simulation. It is suggested that although this approach results in less realistic jet morphologies and significant inaccuracies in the vicinity of the jet, these inaccuracies should not profoundly affect our predictions of free-surface velocity, which are made some distance away from the jet. One final note: the reader should recognize that the meshes shown in Figure 4 and Figure 5 are far too coarse for realistic predictions and were created merely for demonstration purposes: to demonstrate the utility of employing a gradation in mesh density. To be perfectly clear as to the graded meshing strategy, computational cells in the aluminum near the copper might start off looking something like what is depicted Figure 6a and approach something like what is depicted in Figure 6b. This is in opposition to computational cells that start off looking something like what is depicted in Figure 6c and approach something like what is depicted in Figure 6d. Engineering judgement and perhaps trial and error dictates details of the gradation strategy.

### 3.2. Numerical Considerations Related to Impedance Matching

The term impedance is, unfortunately, somewhat context-dependent. Electrical impedance refers the ratio of electrical potential to current and has the units of V/A. Within the context of continuum mechanics, impedance (often, though not exclusively, referred to as acoustic impedance) is the ratio of pressure to displacement for quasistatic or elliptic systems and has the units of Pa/m. For dynamic or hyperbolic systems it is the ratio of pressure to velocity and has the units of Pa-s/m. Within the context of the current work, the governing system of equations is hyperbolic and the relevant impedance, *z*, is defined as z=ρc, where ρ is the density and *c* is the appropriate wave speed (either longitudinal or shear.

(34) $$c=\sqrt{\frac{\mu }{\rho }}\;\mathrm{for}\;\mathrm{shear}\;\mathrm{waves}$$

(35) $$c=\sqrt{\frac{\lambda +2\mu }{\rho }}\;\mathrm{for}\;\mathrm{longitudinal}\;\mathrm{waves}$$

where λ and μ are the Lamé constants.

The modeler (whether using finite element, finite volume, or perhaps some other numerical method) should consider impedance, a priori, for achieving an accurate solution to the relevant initial boundary value problem (IBVP) may require impedance matching at material interfaces. This is because without it, under certain circumstances, spurious non-physical reflections and/or transmissions may occur [17].

The question of whether impedance matching is required for an accurate solution of the IBVP under consideration in the current study is significant. Consider the ramifications with respect to the HE/Cu interface in light of the “to slide or not to slide” conundrum discussed in Section 3.1.1. As discussed already, if one choses to prevent the copper cylinder from sliding within its aluminum confinement (and ultimately out of that confinement), a consequence is the production of very elongated computational cells in the copper in the vicinity of the aluminum. Moreover, that production is exacerbated if impedance matching at the HE/Cu interface is imposed. The combination of computational cells with very high aspect ratios, combined with exaggerated curvilinearity in the copper that results from partially fixing the nodes at z=0, significantly increases the likelihood of tangling within the Cu. That being the case, it would be convenient to forego impedance matching at the HE/Cu interface. This is because impedance matching will result in cells with a much higher aspect ratio (and propensity for tangling) than is the case without enforcing an impedance match. Fortunately, it is rather easy to demonstrate that such foregoance results in negligible innacuracies, especially in the vicinity of the primary diagnostic (PDV probe locations). Larger inacuracies may incur in the vicinity of the Cu/Al interface, but getting the details precisely right in this region is not our focus, rather, we need only assure that any inacuracies that our modeling produces in this region are far enough removed from the diagnostics that the impact at diagnostic locations is negligible.

**Figure 7:**
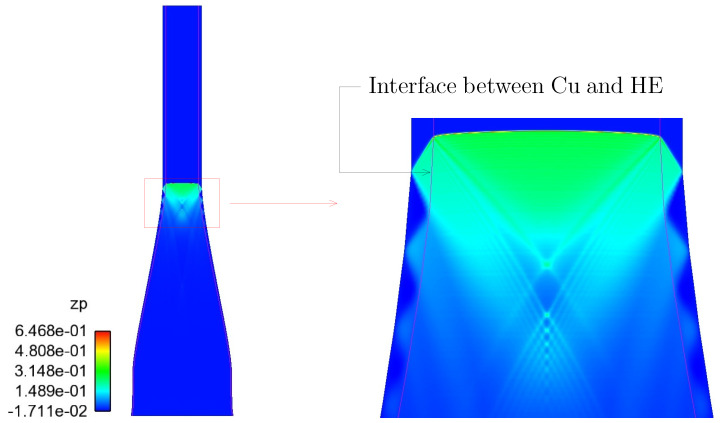
Detonation front at 20 μs.

Let us condider the mechanics of wave propagation with particular focus on the HE/Cu interface. By the time the detonation wave reaches the PDV diagnostics, it is steady. Moreover, the angle of incidence between the longitudinal detonation wave and the HE/Cu interface is rather large (practically 90${}^{\circ }$) as shown in Figure 7. Thus, the detonation wave imparts only a “glancing blow” at this interface. This is fortunate with respect to the need for impedance matching because it serves to minimize that need (errors incurred by failing to impedance match are very small, at least in the vicinity of the diagnostics). This is rather easily verified numerically by conducting a series of simulations starting with an enforcement of impedance matching at the HE/Cu interface and comparing those results to others obtained from simulations wherein the impedance matching paradigm is relaxed.

**Figure 8:**
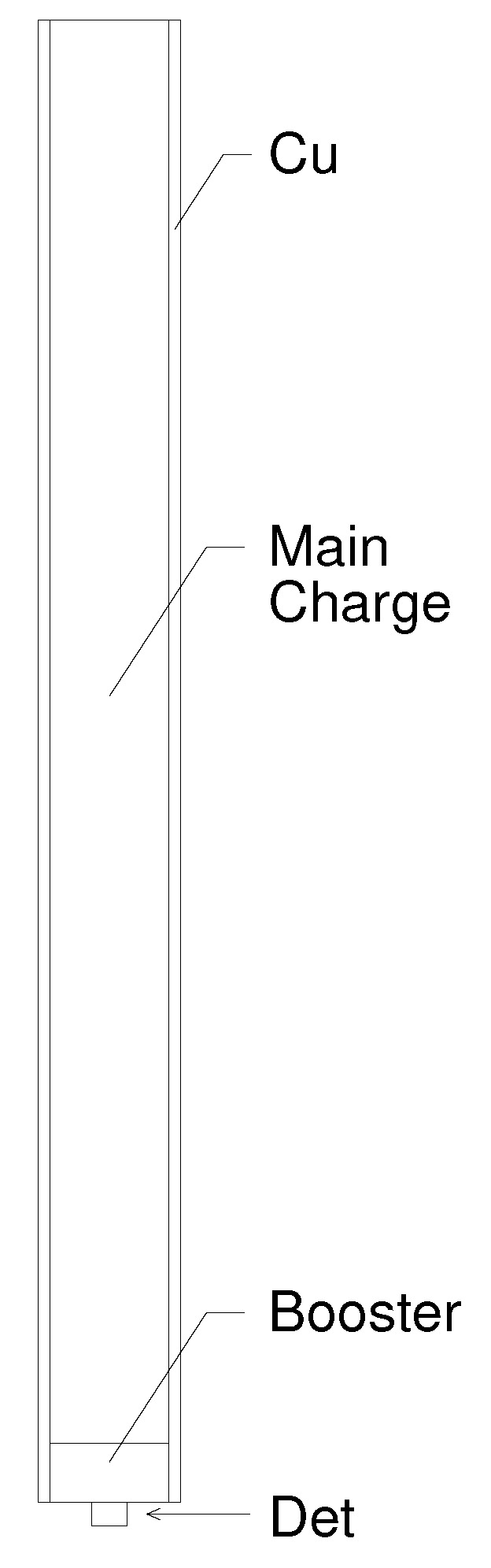
Notional addembly with no aluminum plates.

To conduct this series of simulations, the authors have chosen to model a simplified/notional experiment, as opposed to the actual experiment. A diagram of the notional experimental assembly is provided in Figure 8. This notional experiment has neither upper nor lower aluminum plate. Consequently, our modeling of this notional experiment is necessarily innacurate in regions of space near the lower aluminum plate. This is true for all time (commencing with detonation initiation, which occurs at t = 0, and continuing through to the end of simulation). Omission of the upper aluminum plate incurs minimal inacuracies (if any at all) until late time, late enough that any impact that it may have has no bearing on our assessment of the need for impedance matching, which is our current focus. We argue that our modeling of the notional experiment to address the question of impedance matching can be justified because in this aspect of the analysis we are only concerned with wave propagation well removed from the lower aluminum plate, in regions where the detonation front is steady and the angle of incidence at the HE/Cu interface is almost 90${}^{\circ }$. The results would be much less valid near *z* = 0 (see Figure 1).

**Figure 9:**
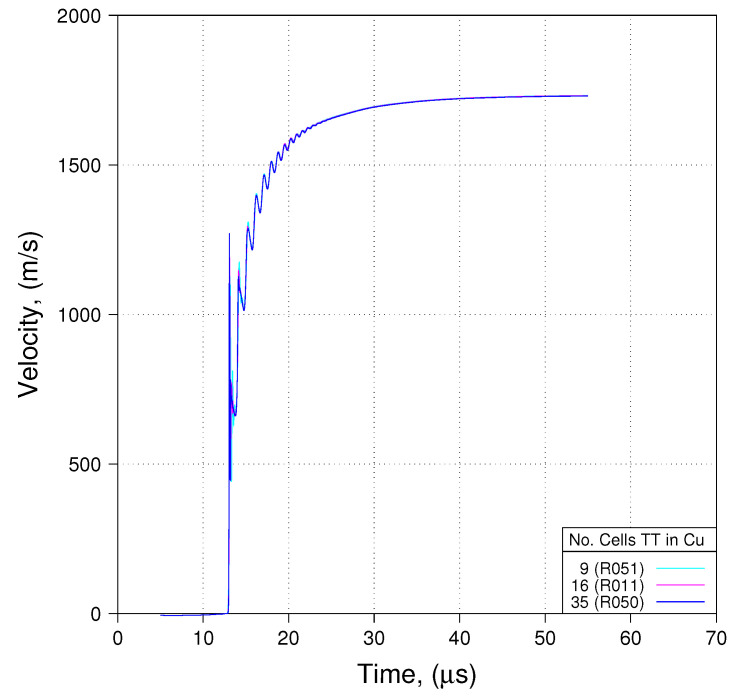
Impedance matching is unnecessary (PDV traces Probe 1).

Impedance matching at the HE/Cu interface dictates 35 computational cells within the Cu as counted in the through-thickness (TT) direction. Simulations were conducted with 35, 16, and 9 cells within the Cu as counted in the TT direction. Figure 9 depicts the results from these three simulations. What is shown in Figure 9 is predicted free surface velocity at the location of PDV Probe 1. Note that excepting some minor differences that show up early on (during the first “ring”) all three predictions fall one-on-top-of-another, providing clear confirmation that with respect to comparisons to the primary diagnostic, impedance matching is not required at the HE/Cu interface.

### 3.3. Numerical Considerations Related to ALE

An additional numerical consideration that one must face in modeling the high explosive cylinder experiment has to do with the question of ALE, and how much of it to employ. When run in pure Lagrange mode FLAG is conservative in total energy. Moreover, in this case (i.e., pure Lagrange) the breakdown of total energy into its component parts is acceptably accurate. However, during the conduct of this work it has become evident that once ALE is employed, the accuracy of energy balance suffers. This is manafest in the modeling of the HE cylinder experiment as an artificial loss in kinetic energy of the Cu cylinder (the greater the use of ALE, the greater the loss). Knowledge of this is of paramount importance, for if one is to have any hope of modeling the experiment including the lower aluminum plate, a lot of ALE will be required.

**Figure 10:**
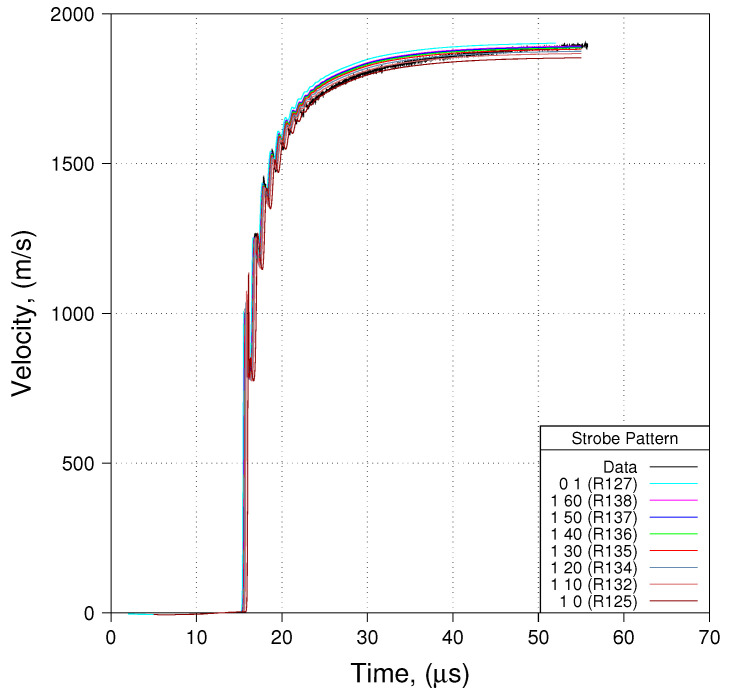
Impact of arbitrary Lagrangian–Eulerian (ALE) (Shot 01, Probe 07, Res 200 μm).

To illustrate the magnitude of the difficulty, the authors have conducted a series of simulations using the simplified notional geometry (discussed previously) wherein the aluminum plates are completely absent. Use of this notional geometry is most probably insufficient and inaccurate to some degree in modeling the integrated HE Cylinder test, but is informative if one wishes to focus only on the intermediate time (the time within which PDV measurements are relevant) impact of ALE, which is our current focus. To assess that impact, a series of simulations have been conducted with varying degrees of ALE, ranging from pure Lagrange to full ALE. By full ALE we mean ALE involving nodal relaxation on every time step once relaxation commences. The impact of ALE is illustrated in Figure 10. In this figure, simulated free surface velocity is compared to empirical data for runs involving varying amounts of ALE. The amount of ALE that is employed is controlled by on/off strobing of nodal relaxation. Eight separate simulations were conducted. The results of these eight simulations are distinguishable in Figure 10 by the pattern of strobing: 1 60 denotes nodal relaxation on for one timestep then off for 60, 1 50 denotes nodal relaxation on for one timestep then off for 50, and so on. A strobing pattern of 0 1 denotes pure Lagrange and a strobing pattern of 1 0 denotes full ALE, i.e., nodal relaxation on every time step once relaxation commences.

Let us contemplate a few observations that can be made concerning Figure 10 and the impact of ALE. First, the simulation result that matches the data most closely is that for which the strobing pattern is 1 60. Second, with increasing ALE we observe increasing loss of kinetic energy in the copper cylinder. Third, while the simulation results obtained from the pure Lagrange run are reasonably close to the empirical data, the results obtained with a strobing pattern of 1 60 are actually closer. This suggests that in order to maintain acceptable mesh quality some degree of ALE is absolutely required.

In conclusion to the question of ALE and how much to employ, it appears that the best strategy may be to employ a minimal amount: just enough to maintain a reasonably smooth mesh, but not so much as to artificially damp out kinetic energy. Finally, these observations are only valid if our modeling of the notional experiment (as opposed to the actual experiment) can indeed reflect accurately the impact of ALE. We would argue that this is the case so long as we focus solely on a region of space that is sufficiently far from the lower aluminum plate. Since our analysis is based upon behavior at the location of PDV Probe 7, our conclusions are quantitatively accurate only to the degree that the location of Probe 7 is sufficiently far removed from the lower plate that the impact of the lower aluminum plate is negligible in so far as free-surface velocity is concerned. Whether this is the case, or not, will be addressed further a little latter in this paper.

The observations just discussed have profound implications for modeling the experiment at hand. We can not model the experiment accounting for the copper/aluminum interaction without using a lot of ALE. If we use a lot of ALE we loose (artificially) a significant amount of kinetic energy in the Cu cylinder. This conundrum leads naturally to the following thought experiment. Let us suppose that the HE/Cu cylinder is very long, perhaps much longer than the actual composite cylinder. In this case, it is self-evident that at some value of *z*, $z=z_{c}$, the impact of the lower aluminum plate becomes vanishingly small, and at any value of $z>z_{c}$, the impact of the lower aluminum plate is null and neglecting its presence all together is justified. In this case (that is, $z>z_{c}$), modeling of the notional experiment as opposed to the actual experiment is fully justified and the numerous difficulties that arise from presence of the lower aluminum plate are eliminated. The question as to the value of $z_{c}$ will be addressed later in this paper.

### 3.4. Another Numerical Consideration Related to the Lower Aluminum Plate: $z_{c}$

**Figure 11:**
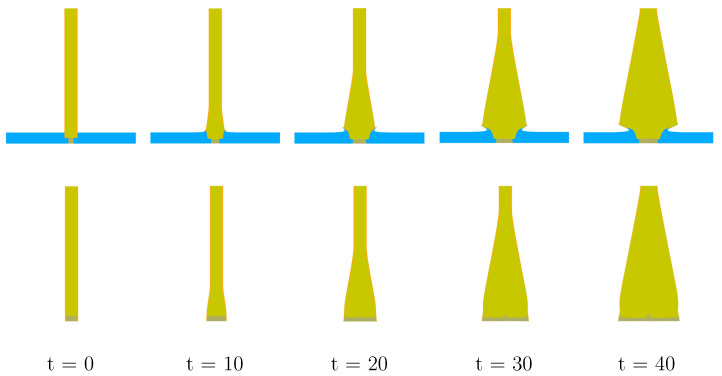
With and without lower aluminum plate (all times in μs).

Based upon the thought experiment discussed in Section 3.3 we are justified in modeling the simplier notional experiment so long as $z_{p}>z_{c}$ ($z_{p}$ references the locations of the primary diagnostic—see Figure 1). Let us now explore whether that is the case in the experiments under study. To conduct this exploration the authors have conducted simulations with and without the lower aluminum plate being present. Representative comparisons of the resultant geometric morphologies at several points in time are provided in Figure 11. It is obvious that in the vicinity of the lower aluminum plate the differences in resultant morphology are extreme. However, it is not immediately obvious that the differences are significant at the location of the PDV diagnostics, about half-way up the cylinder. Are the differences at PDV probe locations significant? To seek an answer to this question, let us consider predicted free-surface velocity traces.

**Figure 12:**
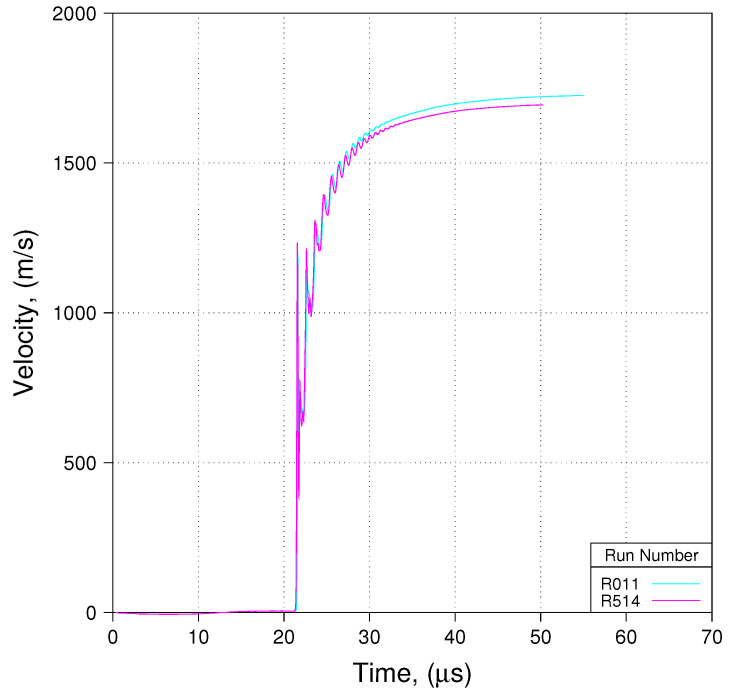
Impact of presence/absence of lower aluminum plate (Shot 01, Probe 08, Res 158.75 μm).

Figure 12 provides traces of predicted free-surface velocity at the location of Probe 8, the probe location with the greatest value of *z* (*z* = 17.80 cm, or 15.40% of *L*, where *L* = 30.38 cm). Simulation R514 included the lower aluminum plate, simulation R011 did not. Casual observation concerning Figure 12 reveals that while perhaps not large, the difference between the two predictions is significant. Consequently, one must, unfortunately conclude that in this particular set of experiments one should probably account for the presence of the lower aluminum plate. If the PDV probe locations had been farther removed from the lower aluminum plate, the authors hypothesize that differences between results obtained accounting for the lower aluminum plate and those obtained from simulations wherein the plate is absent would trend toward zero as *z* increases. We will have more to say on this matter latter in this paper.

One final comment on the conclusions derived from observations concerning Figure 12 is in order. Conducting simulations of the actual experiment, accounting for the presence of the lower aluminum plate is difficult, and the difficulty increases with increasing mesh density. The average cell size in R514 and R011 was approximately 160 μm. This resolution represents a mesh that is far too coarse for precise evaluations of constitutive behavior and of reactive burn, which is typically the main purpose for conducting HE cylinder expansion tests. Ideally, we would like to conduct the simulations with a mesh that has a resolution of 100 μm or smaller. Depending upon the thermophysical behavior or mechanism that one seeks to evaluate, mesh resulution may need to be considerably finer than 100 μm. In the present work we found that keeping the simulation from crashing early with a mesh that is much finer than 160 μm requires Herculean effort, and is, in most cases, simply not practical. Consequently, either a way must be found to enable robust simulation of the actual experiment using meshes with sufficiently fine resolution, or a way must be found to justify use of the notional experimental assembly, for which going to very fine mesh resolution poses no great difficulty. We shall expound upon this conundrum latter in this paper.

### 3.5. Numerical Considerations Related to Detonation Velocity

In light of what has preceeded, the reader will see in what follows that numerical considerations related to a determination of detonation velocity are far from straightforward. We have seen that to obtain the most accurate prediction of free-surface velocity, we need to employ ALE, but a minimal level only. We have also seen that if we ignore the lower aluminum plate (i.e., model the notional experiment as opposed to the actual experiment) we incurr significant error because $z_{p}<z_{c}$ in the set of experiments under consideration here. With these two observations in mind, let us consider detonation velocity.

**Figure 13:**
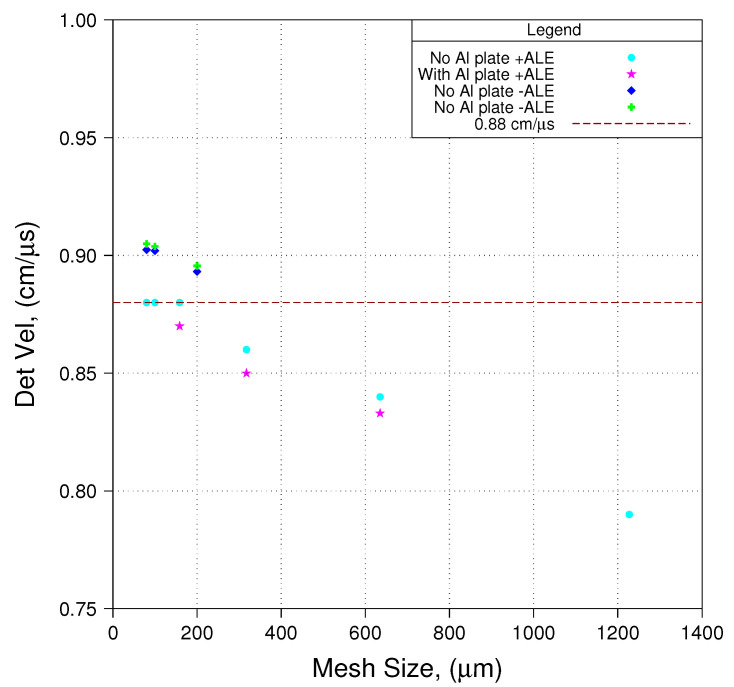
Detonation Velocity.

We have conducted a number of simulations with a wide range in mesh resolution (average cell size ranging from 80 to ∼1200 μm). In some of these simulations the actual experiment was modeled, in others the notional experiment was modeled. Figure 13 compares predicted detonation velocity to its accepted value for PBX 9501, which is ∼0.88 cm/μs. Results labeled +ALE refer to simulations conducted in full ALE mode, that is, ALE on every time step once ALE commences, which was rather early in the simulations conducted for this work: 1 μs. Results labeled -ALE refer to simulations conducted using minimal ALE. Note that the only difference in the two -ALE results shown is that differing sets of flow stress parameters were used for Cu, the impact of which is minimal. We observe that if we model the notional experiment and use full ALE, predicted detonation velocity approaches the accepted value from below and matches the accepted value so long as average cell size is about 160 μm or smaller. However, since $z_{p}<z_{c}$ in this set of experiments, we know that modeling in this manner incurs significant error (see Figure 10) with respect to predictions of free-surface velocity. Similarly, if we model the actual experiment and use full ALE, predicted detonation velocity approaches the accepted value from below and extrapolation would suggest that at a resolution of approximately 80 μm or finer, predicted detonation velocity is likely to match the accepted value. However, as stated earlier, we have found that to model the actual experiment using mesh resolution finer than about 160 μm requires Herculean effort. We observe that if we model the notional experiment and use minimal ALE (as required to obtain the best prediction of free-surface velocity) predicted detonation velocity is greater than the accepted value, and in fact diverges from the accepted value with increasing mesh resolution, at least for resolutions down to 80 μm. We will have more to say on detonation velocity a little later in this paper.

### 3.6. Numerical Considerations Related to Artificial Viscosity

In addition to the previously discussed numerical considerations of modeling the HE cylinder expansion test, there are a number of nuanced issues that the modeler should consider that are related to artificial viscosity. We delay a thorough discussion of those to a later date.

## 4. An Evaluation of the Proposed Phenomenological Parameter Sets

**Figure 14:**
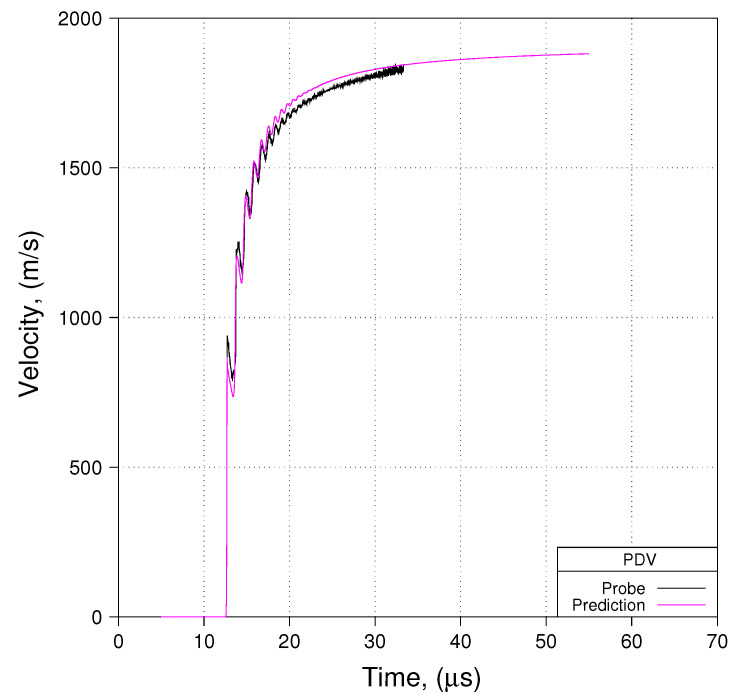
Prediction to Data: Shot 01, Probe 01, R165.

**Figure 15:**
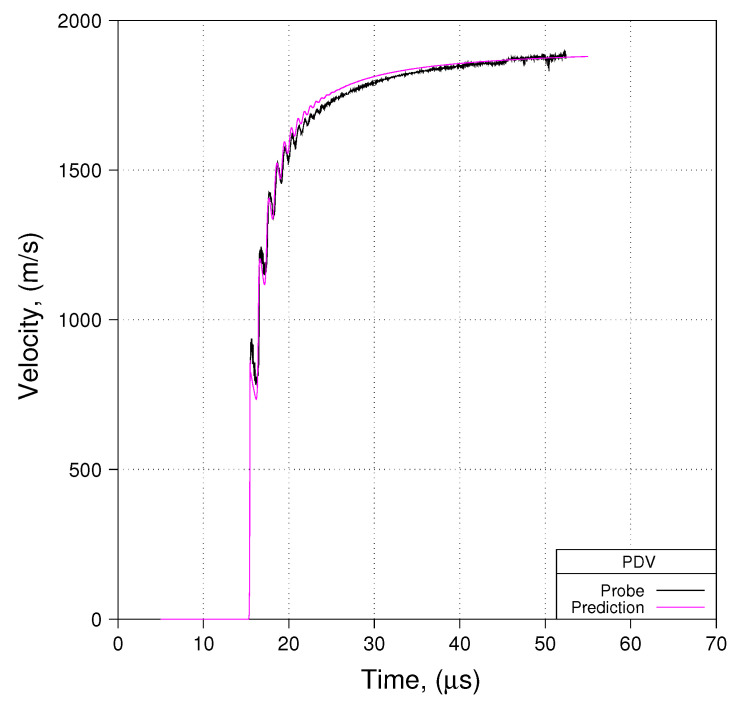
Prediction to Data: Shot 01, Probe 02, R165.

**Figure 16:**
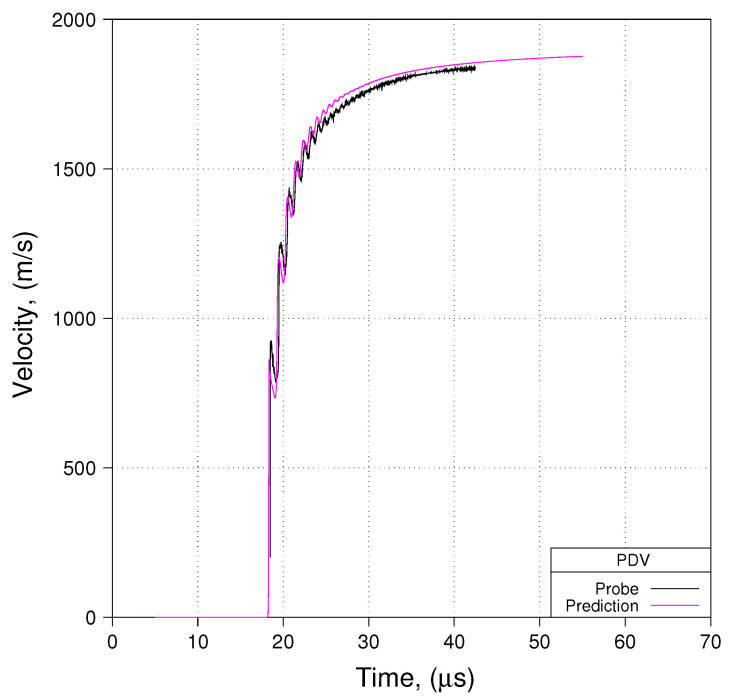
Prediction to Data: Shot 01, Probe 03, R165.

Having considered some of the numerical considerations that the modeler must ponder, let us turn our attention to an evaluation of the proposed phenomenological parameter sets presented earlier (for Davis-type EOSs and for WSD reactive burn). We shall base this analysis upon use of the notional experiment using minimal ALE and meshes with an average cell size of 80 μm. Comparisons of predicted free-surface velocity to measured free-surface velocity are presented in Figure 14, Figure 15 and Figure 16. Figure 14, Figure 15 and Figure 16 show comparisons for Shot 01 at probe locations 1, 2, and 3. Comparisons for Shots 4 and 6 look very similar to what is shown in Figure 14, Figure 15 and Figure 16.

We see in these three Figure 14, Figure 15 and Figure 16 that predicted free-surface velocity matches measured free-surface velocity reasonably well. However, let us impose a few caveats onto that statement. First, we have shown that $z_{p}$ in these experiments is less than $z_{c}$. Conseqently, by basing our evaluation of phenomenological parameter sets on modeling the notional experiment as opposed to the actual experiment, we accept error in predicted free-surface velocity with an order of magnitude such as is suggested in Figure 12. Second, by modeling the notional experiment using minimal ALE, we accept error in the prediction of detonation velocity that, as suggested in Figure 13, is on the order of 2% ( 0.90 vs. 0.88 cm/μs).

One final note. We have recently developed other parameter sets, some of which represent improvements over the set evaluated here. A portion of that work has been discussed briefly elsewhere (see [18,19]). We expect to provide a more thorough exposition on that topic in the near future.

## 5. Summary Discussion of Results and Implications for Future Work

As discussed earlier, presence of the lower aluminum plate results in jetting, along with other phenomena, that lead to numerical difficulties. Allowing the copper cylinder to slide within the confines of the lower aluminum plate (ultimately escaping that confinement) leads to numerical difficulties. Not allowing the copper cylinder to slide within the lower aluminum plate (leading to localized extrusion at the lower end of the copper cylinder resulting in elements with very high aspect ratio and a propensity for tangling) leads to numerical difficulties. It has been shown that impedance matching at the HE/Cu interface is not required, which is fortunate indeed for its inclusion would serve to exacerbate the aspect ratio and tangling problem.

Numerical difficulties make modeling the experiment in FLAG using a mesh of sufficiently fine resolution a rather challenging undertaking. Based upon the current work, we propose that a reasonably accurate prediction of free-surface velocity requires mesh resolution such that the average cell size is on the order of 100 μm or smaller. Attaching to this proposition the observation that modeling the experiment with a mesh resolution finer than 160 μm requires Herculean effort, one is faced with what seems, at first glance, to be an untenable challenge. However, as is often the case in such situations, with an application of engineering judgment resulting in the adoption of simplifications, the untenable becomes the approximate yet achievable. A simplification that removes many of the aforementioned numerical difficulties involves removal of the lower aluminum plate from consideration, thereby modeling the notional experiment as opposed to the actual experiment. This is the approach that was ultimately adopted in the current work for our evaluation of constitutive and reactive burn phenomenological parameter sets. The authors wish to emphasize at this point that adopting this simplification is nothing new, in fact, it is rather the norm in the modeling of HE cylinder expansion tests. At this point the reader might be tempted to ask: if the norm is to ignore the aluminum plates why did the authors not adopt this simplification from the outset. The reason is that the authors suspected *a priori* that the PDV probes in this particular suite of expermients might be too close to the lower aluminum plate ($z_{p}<z_{c}$) resulting in the need to account for the presence of the aluminum plate in order to achieve accuracy in our numerical predictions. Ultimately that suspicion was borne out and the magnitude of the error incurred by ignoring the lower aluminum plate has been herein estimated.

While adopting use of the notional experimental assembly made running the simulations with mesh resolutions sufficiently fine that predictions of free-surface velocity may be considered reasonably accurate, predictions of detonation velocity in the current work are in error by about 2% (about 0.90 cm/μs versus 0.88 cm/μs).

The present work makes clear a number of avenues of endeavor that may prove to be valuable in future work. While the authors have demonstrated that $z_{p}<z_{c}$ in the suite of experiments used for the current work, we strongly suggest that this is not necessarily the case in many other HE cylinder expansion tests that have been conducted over the years. Moreover, this work begs the question as to what is the value of $z_{c}$. We feel strongly that providing the experimental and modeling communities with guidance as to the value of $z_{c}$ will prove to be a highly valuable piece of information. We have undertaken this determination and plan to publish our findings in the very near future. We have shown that it is best to model the experiment using minimal ALE. We have also shown that if we use minimal ALE we over-predict detonation velocity by about 2% in simulations of the notional experiment. Moreover, we have shown that as mesh resolution increases (e.g., from 200 to 100 to 80 μm—see Figure 13) predicted detonation velocity diverges from the accepted value. The authors have explored this phenomenon rather carefully and have found that as mesh resolution increases (e.g., beyond 80 μm), predicted detonation velocity continues to diverge until a critical mesh density is reached. Once that critical mesh density is attained, predicted detonation velocity drops to the accepted value and remains there with ever increasing mesh resolution. The authors are finalizing their investigations of this phenomenon and plan to publish those findings, along with an explanation of those findings, in the very near future. The authors found that modeling the actual experiment with a mesh resolution finer than about 160 μm to be impractical. However, the FLAG code is under continual development and it is quite likely that some recently incorporated capabilities related to remapping will significantly improve this situation. It should be noted that the authors employed nodal relaxation without full remapping in the current work. The authors plan to carefully explore these recently incorporated remapping capabilities in our work aimed at a determination of $z_{c}$. It is also possible that work being done to endow FLAG with adaptive mesh refinement (AMR) capabilities will improve matters in this regard. It has been suggested that there may be some benefit to using triangular (as opposed to quadrilateral) computational cells in the simulation, but FLAG is taylored to the use of quads and as such this supposition will have to be tested at a future date using some other simulation code. One final point that we would like to make relates to error. We have on numerous occasions referred to error in our numerical simulation without quantifying that error. This is because, in its present state, FLAG does not incorporate error estimation. This could be accomplished by adding the calculation of an appropriate mathematical norm, such the energy or L2 norm, to the suite of FLAG modules, but unfortunately, as of today, this capability is simply not available.

## 6. Conclusions

A series of experiments involving the detonation of PBX 9501 encased in a copper cylinder have been modeled with the objective of evaluating a set of phenomenological parameters for Davis-type EOSs and the WSD reactive burn model. Perhaps running counter to initial impressions, the authors have found the modeling of this suite of experiments using the FLAG code to be rather challenging. Specifically, we found that modeling in sufficiently high resolution without introducing simplifications that result in numerical error to be a non-trivial matter.

We have shown that if we model the simplified notional experiment, as opposed to the actual experiment, and employ meshes with a resolution ≤ 100 μm, that we are able to match the primary diagnostic, PDV measurements of free-surface velocity, reasonably well. However, we have also noted a number of errors that have been absorbed into our analysis. For example, error induced from not accounting for the impact of the lower aluminum plate, and error in predictions of detonation velocity resultant from using an insufficiently fine mesh. That being the case, we conclude that the evaluated set of phenomenological parameters is at least reasonable, but that further work is required. The authors have conducted and are continuing to conduct additional work in this area and plan to present a more thorough evaluation and quantifiable justification of phenomenological parameter sets in the near future. 

## Figures and Tables

**Table 1:** Axial coordinate and incidence angle α for each of the eight Photon Doppler Velocimetry (PDV) probe spots.

Probe	Shot 1		Shot 4		Shot 6	
	*z*, (cm)	α, (deg)	*z*, (cm)	α, (deg)	*z*, (cm)	α, (deg)
1	9.66	3.970	9.46	7.028	9.76	4.731
2	12.16	4.355	12.06	7.028	12.29	4.927
3	14.76	3.950	14.86	5.088	14.86	4.338
4	17.13	5.117	17.08	5.477	17.08	5.515
5	11.91	7.054	12.11	7.316	11.96	6.726
6	17.13	5.082	16.88	6.934	16.93	6.715
7	12.06	5.959	12.16	6.404	12.06	6.597
8	17.80	4.790	17.18	4.837	16.98	6.212

**Table 2:** Davis reactants parameter set.

Parameter	Description	Value
*A*	Param. in Equation (6)	0.21 $\left(\mathrm{cm}\right){\left(\mu s\right)}^{-1}$
*B*	Param. in Equations (3) and (6)	3.8
*C*	Param. in Equation (3)	0.4
$\alpha_{st}$	Param. in Equation (2)	0.3662
$C_{v0}$	Param. in Equation (2); (Spec. heat at ref. density)	10.67 × $10^{-6}$ $\left({\mathrm{Mbar}\;\mathrm{cm}}^{3}\right)({\mathrm{gK})}^{-1}$
$\Gamma_{0}$	Param. in Equations (5) and (8); (Grüneisen gamma at ref. density)	0.67
$\rho_{0}$	Param. in Equations (6)–(8); (initial (*ref.*) density)	1.832 $\left(g\right){\left({\mathrm{cm}}^{3}\right)}^{-1}$
$T_{0}$	Param. in Equation (8); (initial (*ref.*) temperature)	297 K
*Z*	Param. in Equations (5) and (8)	0.0

**Table 3:** Davis products parameter set.

Parameter	Description	Value
*a*	Param. in Equations (11), (12) and (14)	0.867302
*b*	Param. in Equation (13)	1.04
$C_{vp}$	Param. in Equation (10); (Spec. heat at ref. density)	4.34 × $10^{-6}$ $\left({\mathrm{Mbar}\;\mathrm{cm}}^{3}\right){\left(\mathrm{gK}\right)}^{-1}$
$e_{0}$	Param. in Equation (12)	0.0566997 $\left({\mathrm{Mbar}\;\mathrm{cm}}^{3}\right){\left(g\right)}^{-1}$
*k*	Param. in Equations (11) and (12); (limit of adiabatic gamma at large vol.)	1.33
*n*	Param. in Equation (14)	1.00164
$p_{c}$	Param. in Equation (15)	0.0282 Mbar
$\nu_{c}$	Param. in Equations (11), (12), (14) and (15)	0.95 $\left({\mathrm{cm}}^{3}\right){\left(g\right)}^{-1}$

**Table 4:** Wescott–Stewart–Davis (WSD) parameter set.

Parameter	Description	Value
$r_{a}$	Param. in Equation (28)	0.214
$r_{b}$	Param. in Equation (28)	0.9562
$r_{c}$	Param. in Equations (29) and (30)	0.9562
$r_{d}$	Param. in Equations (29) and (30)	0.0
$r_{e}$	Param. in Equation (31)	0.9562
$r_{g1}$	Param. in Equation (29)	6377.78 ${\left(\mu s\;{\mathrm{Mbar}}^{3.3898}\right)}^{-1}$
$r_{g2}$	Param. in Equation (31)	6377.78 ${\left(\mu s\;{\mathrm{Mbar}}^{3.3898}\right)}^{-1}$
$r_{i}$	Param. in Equation (28)	0.0 μs${}^{-1}$
$r_{k}$	Param. in Equation (30)	6377.78 ${\left(\mu s\;{\mathrm{Mbar}}^{3.3898}\right)}^{-1}$
$r_{n}$	Param. in Equation (30)	3.3898
$r_{x}$	Param. in Equation (28)	0.0
$r_{y}$	Param. in Equation (29)	3.3898
$r_{z}$	Param. in Equation (31)	3.3898
$\rho_{0}$	Param. in Equation (28)	1.844 $\left(g\right){\left({\mathrm{cm}}^{3}\right)}^{-1}$
$\rho_{c}$	Param. in Equation (28)	2.740 $\left(g\right){\left({\mathrm{cm}}^{3}\right)}^{-1}$
$r_{\mathrm{switch}}$	Param. in Equations (29)–(31)	0.90
rkdead	Rate const. in preshock desensization	0.0 μs${}^{-1}$
rpign	Min. pressure to start burn	1 × $10^{-5}$ Mbar
rpmax	Max. pressure at which to deaden	0.06 Mbar
rphel	HEL Min. to sesensitize	0.0007 Mbar
